# Epidemiological situation of bovine tropical theileriosis in an arid region in central Tunisia with a phylogenetic analysis of *Theileria annulata*

**DOI:** 10.1002/vms3.1276

**Published:** 2023-09-19

**Authors:** Khawla Elati, Ismail Salhi, Ridha Kodia, Mourad Rekik, Mohamed Gharbi

**Affiliations:** ^1^ Institute of Parasitology and Tropical Veterinary Medicine Freie Universität Berlin Berlin Germany; ^2^ Veterinary Centre for Resistance Research, Department of Veterinary Medicine Freie Universität Berlin Berlin Germany; ^3^ Laboratoire de Parasitologie École Nationale de Médecine Vétérinaire de Sidi Thabet, Institution de la Recherche et de l'Enseignement Supérieur Agricoles. Univ. Manouba Sidi Thabet Tunisia; ^4^ International Centre for Agricultural Research in the Dry Areas (ICARDA) Amman Jordan

**Keywords:** cattle, Giemsa, PCR, *Theileria annulata*, Tunisia

## Abstract

**Background:**

Tropical theileriosis, *Theileria annulata* infection, is the most prevalent summer disease in Tunisia. It is transmitted by *Hyalomma scupense*, a two‐host tick known to be endophilic.

**Objectives:**

The present study aimed to estimate the infection prevalence of cattle by *T. annulata* in two districts from central Tunisia.

**Methods:**

Blood samples collected from 270 Holstein cattle from the Sidi Bouzid (140 samples) and Kasserine districts (130 samples) were analysed by Giemsa staining and *T. annulata*‐specific PCR.

**Results:**

In both regions, PCR revealed a prevalence of 32.6%. This was significantly higher than the 6.3% prevalence obtained by Giemsa staining blood smears (*p* < 0.001). Giemsa staining also revealed a low parasitaemia of 0.05%. The PCR‐based prevalence was not statistically different between the two districts (31.4 ± 0.04 and 33.8 ± 0.04% in Sidi Bouzid and Kasserine districts, respectively, *p* = 0.6). On the contrary, the results of blood smear examination (2.85 and 10% in Sidi Bouzid and Kasserine, respectively) differed significantly between the two sampling sites (*p* = 0.01). There was no evidence of a statistically significant difference between the overall molecular infection prevalence when the samples were segregated based on animals’ age or gender (*p* = 0.1 and 0.2, respectively) and a similar trend was observed for Giemsa staining. Ten PCR amplicons of the Tams1 gene (721 bp) were subsequently sequenced from the two regions. The phylogenetic analyses showed 100% similarity between all sequences. The unique conserved Tams1 sequence was deposited in GenBank under the accession number OP428816 and used to infer its phylogenetic relationships with those available in the GenBank repository.

**Conclusions:**

This is the first report of the presence of *T. annulata* in this region of central Tunisia which has no history of tropical theileriosis. Priority areas for future studies include understanding the origin of these *T. annulata*‐positive animals in a region where the presence of a known natural vector tick, *H. scupense*, has not been reported. Given that the disease severely constrains cattle productivity, it would also be worthwhile to investigate if other potential vectors for *T. annulata*, such as *Hyalomma dromedarii*, are present in the arid regions.

## Graphical abstract

- *Theileria annulata* is reported for the first time in two arid regions in central Tunisia.
- Infection prevalence of cattle by *T. annulata* was high: Kasserine (33.8%) and Sidi Bouzid (31.4%).
- Phylogenetic analyses of amplicons showed 100% similarity and one unique conserved sequence.

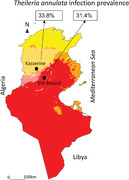

## 1 INTRODUCTION

With a population of 660,300 heads, cattle breeding is a key component of agricultural production in Tunisia and is essential to the economy (Tunisian Ministry of Agriculture, 2016). It generates three vital products: milk, meat and leather. Unfortunately, several constraints threaten the cattle industry, key among these being poor livestock management and animal health issues. In Tunisia, the parasitic leukoproliferative disease, tropical theileriosis (TT) is the most prevalent summer disease in Tunisia. It is transmitted by *Hyalomma scupense*, a two‐host tick known to be endophilic (Bouattour et al., 1996; Darghouth et al., 1996; Gharbi & Darghouth, 2014).

TT is caused by a protozoan, *Theileria annulata*. Schizonts are the most pathogenetic stage, as they transform leukocytes, inducing their continuous, uncontrolled proliferation (von Schubert et al., 2010). The schizonts develop into piroplasms that infect erythrocytes, and can subsequently be picked up by feeding ticks (Liu et al., 2022).

In Tunisia, TT occurs mainly in the north and central areas of the country, corresponding to three bioclimatic zones: humid, sub‐humid and semi‐arid (Darghouth et al., 1996). In northern part of the country, TT has been mainly reported in Beja district, located in the north‐western part, with a prevalence of 27.4% (80/292) by Darghouth (2000) using the indirect fluorescent antibody test (IFAT). Additionally, in Ariana district, especially in Hessiene region, which is an endemic area of TT, several studies have been conducted over the years, and prevalence using IFAT was reported to vary between 63.9 and 92.8% (Darghouth et al., 2004; Gharbi, 2006). More recently, using PCR, Sallemi et al. (2017) estimated *T. annulata* infection in cattle in the same area at 61% (59/96). Interestingly, co‐infection with *Trypanosoma evansi* was reported in six samples. In another study conducted in Bizerte district, 15.5% of the examined cattle (114/735) were *T. annulata* positive using Giemsa‐stained blood smears (Boussaadoun et al., 2015). *Theileria annulata* has also been reported in farms in Gabes Oasis (south‐eastern Tunisia), with a mean infection prevalence estimated at 26.5% (Gosrani, 1999).

In all the regions surveyed to date, *T. annulata* is present mainly on smallholder farms, which constitute approximately 73% of the total farms in Tunisia (Institut National de la Statistique, 2006). Despite the availability of acaricides for tick control and buparvaquone for chemotherapy, this protozoan still represents an important constraint to the development of the livestock industry in Tunisia. Between 2500 and 3000 TT clinical cases are reported annually in Tunisia (Bahri et al., 1995; Darghouth et al., 2004). A study by Darghouth et al. (2004) showed that losses caused by mortality were estimated to be 4,97,492 Tunisian dinars (142.85 euros) for each death case, which is much higher nowadays as a cow costs at least 900 euros. In contrast, the cost of the theilericidal drug was estimated to be 100 Tunisian dinars per adult animal (34 euros). Gharbi et al. (2006) estimated the losses due to decreases in live weight for subclinical cases to be 14.73% per case, with a total cost of 4049.570 TD (2515.32 euros) per case. In carrier lactating cows, the infection causes a persistent mean milk yield decrease of 0.7 litres per day (Gharbi et al., 2015).

Given the economic impact of *T. annulata* infection outlined above, it remains crucially important to undertake regular surveys aiming to assess the emergence of infection in new areas. In pursuit of this objective, the present study aimed to estimate the prevalence of *T. annulata* in two arid regions in Central Tunisia where TT has not been reported before and to compare the prevalence values obtained using both PCR and Giemsa staining. This epidemiological study was followed by a phylogenetic analysis of the *T. annulata* merozoite surface gene (Tams1) amplicons in the Sidi Bouzid and Kasserine districts.

## 2 MATERIALS AND METHODS

### 2.1 Study areas

**FIGURE 1:**
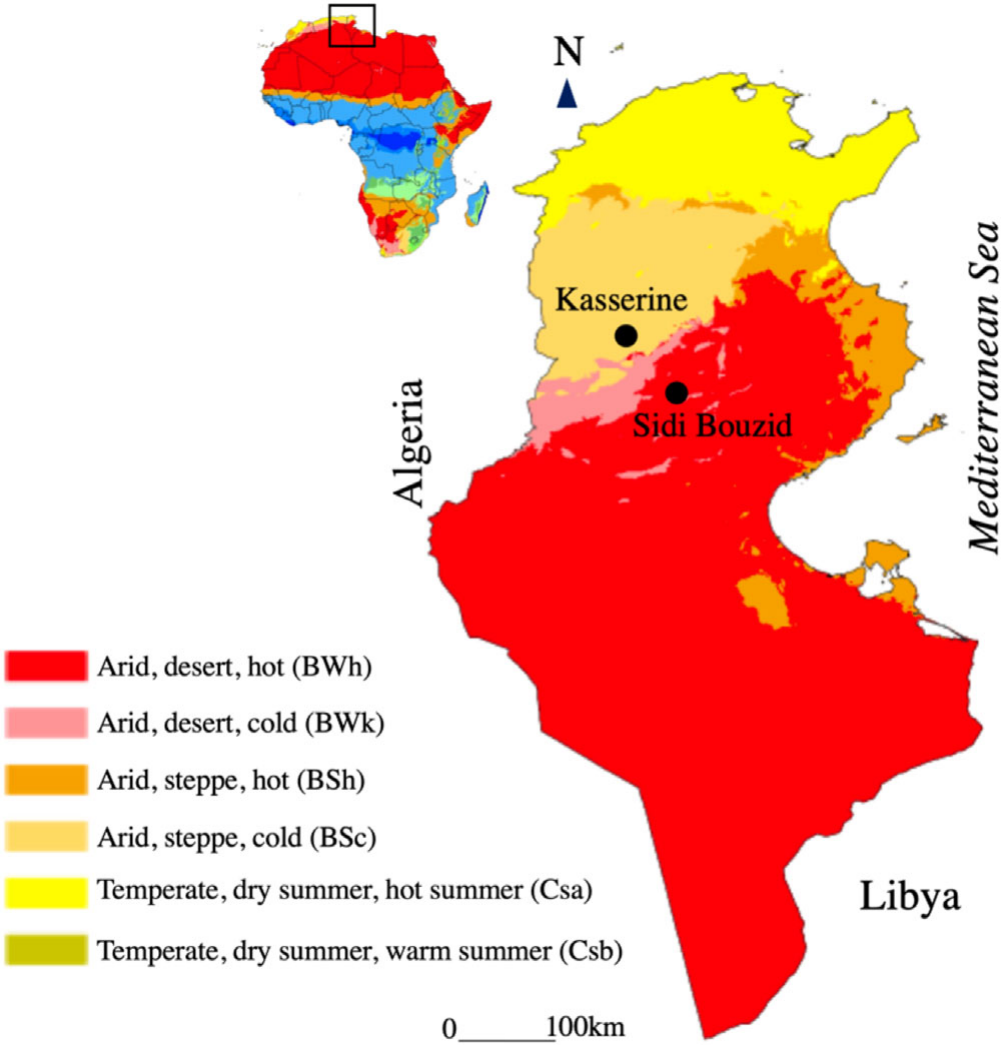
Koppen–Geiger map showing the bioclimatic zones in Tunisia and the location of Sidi Bouzid and Kasserine districts (central Tunisia) (modified from https://en.wikipedia.org/wiki/File:Koppen‐Geiger_Map_TUN_present.svg).

This study was performed in the Sidi Bouzid and Kasserine districts of central Tunisia (Figure 1). Sidi Bouzid has an arid desert climate (BWh climate according to the Köppen–Geiger classification [Peel et al., 2007]), with an annual average temperature of 17.9°C and a mean rainfall of 223 mm. Kasserine district is characterized by an arid steppe climate (BSk climate according to the Köppen–Geiger classification), with a mean temperature of 27.7 and 7.2°C during summer and winter, respectively. The mean annual rainfall is 289 mm (Climate Data.Org). The cattle population in Sidi Bouzid and Kasserine districts was estimated to 61,300 and 14,120 heads, respectively (Tunisian Ministry of Agriculture, 2016).

### 2.2 Animals and sampling

This study enrolled 270 cattle kept under an extensive breeding system. The barns visited were typically poorly maintained, with crevasses and cracks in the walls, and the buildings were mainly constructed from breezeblocks, bricks and stones. Farmers mentioned that they use quicklime to control insects.

The sampled cattle belonged to the Holstein breed and were classified into four age groups: less than 1 year, between 1 and 2 years, between 2 and 4 years and more than 4 years old (Table 1). Blood samples were collected from the jugular vein of each cattle in EDTA‐sterile tubes, and aliquots were stored at −20°C until analysed. No ticks were found on the animals.

**TABLE 1:** Association between *Theileria annulata* infection prevalence in cattle from two districts and the studied parameters.

		Sidi Bouzid				Kasserine				Overall			
		PCR		Blood smears examination		PCR		Blood smears examination		PCR		Blood smears examination	
Parameters		Positive/examined (Prevalence in % ± SE)	*p* Value	Positive/examined (Prevalence in % ± SE)	*p* Value	Positive/ examined (Prevalence in % ± SE)	*p* value	Positive/ examined (Prevalence in % ± SE)	*p* value	Positive/ examined (Prevalence in % ± SE)	*p* value	Positive/ examined (Prevalence in % ± SE)	*p* value
Gender	Male	8/27 (29.62 ± 0.08)	0.8	0	0.3	2/13 (15.4 ± 0.1)	0.1	0/13 (0)	0.1	10/40 (25 ± 0.07)	0.2	0/40 (0)	0.2
	Female	36/113 (31.8 ± 0.04)		4/113 (3.5 ± 0.01)		42/117 (35.9 ± 0.04)		13/117 (11.1 ± 0.03)		78/230 (33.9 ± 0.03)		17/230 (7.4 ± 0.01)	
Age group (years)	[0, 1]	11/36 (30.55 ± 0.07)	0.1	0	0.4	11/35 (31.4 ± 0.08)	0.6	0/35 (0)	0.03^*^	22/71 (30.9 ± 0.05)	0.1	0/71 (0)	0.003^*^
	[1, 2]	6/34 (17.64 ± 0.06)		1/34 (2.94 ± 0.03)		6/23 (26.1 ± 0.09)		2/23 (8.7 ± 0.06)		12/57 (21.05 ± 0.05)		3/57 (5.2 ± 0.03)	
	[2, 4]	17/42 (40.47 ± 0.07)		1/42 (2.38 ± 0.02)		14/33 (42.4 ± 0.08)		3/33 (9.1 ± 0.05)		31/75 (41.3 ± 0.06)		4/75 (5.3 ± 0.02)	
	>4	10/28 (35.71 ± 0.09)		2/28 (7.14 ± 0.05)		13/39 (33.3 ± 0.075)		8/39 (20.5 ± 0.06)		23/67 (34.32 ± 0.06)		10/67 (14.9 ± 0.04)	
	Overall	44/140 (31.4 ± 0.04)		4/140 (2.85 ± 0.01)		44/130 (33.8 ± 0.04)		13/130 (10 ± 0.02)		88/270 (32.6 ± 0.03)		17/270 (6.3 ± 0.01)	0.01

Abbreviation: SE, standard error.

* Significant.

### 2.3 *Theileria annulata* detection

#### 2.3.1 Microscopic examination

Blood smears were made from each animal, Giemsa‐stained and then examined under a microscope at 1000*x* magnification to detect *T. annulata* piroplasms. For each blood smear, 50 microscope fields were examined. The infection intensity by piroplasms, as an indicator of parasitaemia, was calculated as follows:

(1) $$\mathrm{Parasitaemia}\;\;\left(\%\right)=\;\frac{\mathrm{Number}\;\mathrm{of}\;\mathrm{infected}\;\mathrm{erythrocytes}}{\mathrm{Number}\;\mathrm{of}\;\mathrm{examined}\;\mathrm{erythrocytes}}x100.$$

#### 2.3.2 DNA extraction and polymerase chain reaction

DNA was extracted from 300 μL of blood using the BioBasic (Canada Inc.), DNA extraction kit, following the manufacturer's instructions and stored at −20°C until use.

A set of specific primers N516/N517 (forward primer N516: 5′‐GTAACCTTTAAAAACGT‐3′ and reverse primers N517: 5′‐GTTACGAACATGGGTTT‐3) was used to amplify a 721 bp DNA fragment of the *T. annulata* major merozoite surface antigen (Tams1), as described by d'Oliveira et al. (1995).

The PCR was performed in a reaction volume of 25 μL consisting of 2.5 μL of 10× Taq buffer, 3 mM of MgCl_2_ (25 mM)_,_ 0.4 mM of each deoxyribonucleotide triphosphate, 0.5 μM of each primer, 0.25 μL of Taq DNA polymerase and 2 μL of each DNA sample.

The PCR consisted of 30 cycles preceded by an initial 5‐min denaturation step at 94°C, and each cycle had the following steps: denaturation (1 min at 94°C), annealing (1 min at 55°C) and elongation (1 min at 72°C), followed by a final extension at 72°C for 10 min. PCR products were electrophoresed in a 1% agarose gel and then stained with a 1% ethidium bromide solution and the DNA fragments were visualized under UV.

#### 2.3.3 *Theileria annulata* DNA sequencing

A total of 10 positive PCR amplicons were randomly purified and subjected to direct Sanger sequencing in both directions using the same primers used for PCR reactions. The unique sequence obtained was submitted to GenBank under the accession number OP428816. Geneious prime software (v. 2021) was used to assemble the chromatograms, perform sequence alignments and infer phylogenetic relatedness (Kearse et al., 2012).

The *T. annulata* sequence generated in the present study was compared to those available in the NCBI database. The phylogenetic tree was constructed using the neighbour‐joining method (Saitou & Nei, 1987).

#### 2.3.4 Statistical analysis

The infection prevalence was calculated as follows (Bush et al., 1997):

(2) $$\mathrm{Infection}\;\mathrm{prevalence}\;\;\left(\%\right)=\;\frac{\mathrm{Number}\;\mathrm{of}\;\mathrm{infected}\;\mathrm{cattle}}{\mathrm{Number}\;\mathrm{of}\;\mathrm{examined}\;\mathrm{cattle}}x100.$$

A Chi‐square test was performed to assess the statistical relation between prevalence estimated by the two techniques used and the sources of variations: age group and gender, using SPSS software (v. 21, IBM, USA) at a 5% threshold value (Schwartz, 1993).

## 3 RESULTS

### 3.1 *Theileria annulata* infection prevalence of cattle in Sidi Bouzid and Kasserine districts

The microscopic examination of Giemsa‐stained blood samples showed *T. annulata* piroplasms in only 4 out of 140 samples (2.85 ± 0.01%) from Sidi Bouzid and in 13 of the 130 samples (10 ± 0.02%) from Kasserine region (*p* = 0.01). A significant difference by age group was seen for the overall blood smears examined from both districts together (*p* = 0.003) and those obtained from Kasserine only (*p* = 0.03). While, the infection prevalence using blood smear examination from the Sidi Bouzid district showed no significant difference according to age group (*p* = 0.4) (Table 1). The mean parasitaemia in the examined blood smears was 0.05% for both regions.

The molecular detection of *T. annulata* showed that among the 270 analysed cattle samples from the two districts in central Tunisia, 88 were positive by PCR (32.6 ± 0.03%) (Table 1). The molecular infection prevalence was not statistically different between both districts (31.4 ± 0.04 and 33.8 ± 0.04% in Sidi Bouzid and Kasserine districts, respectively [*p* = 0.6]).

**FIGURE 2:**
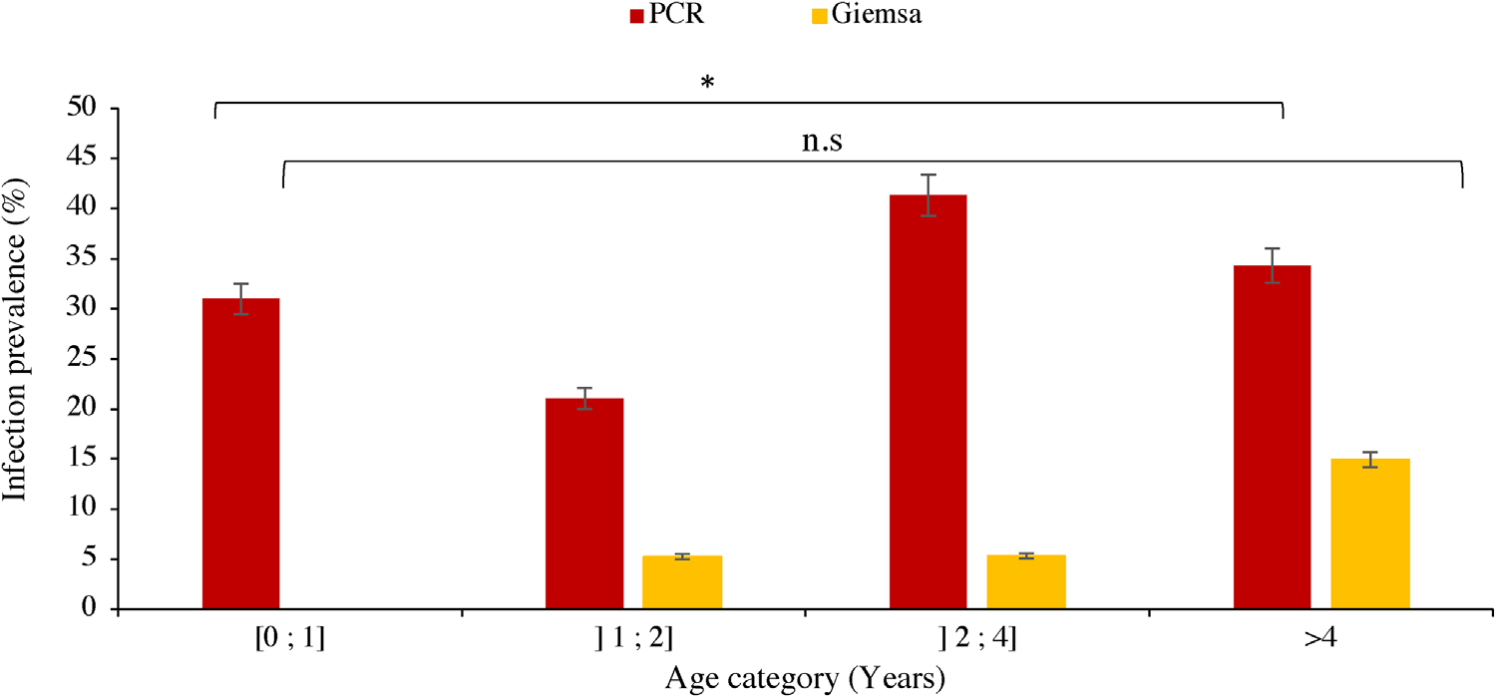
Infection prevalence by *Theileria annulata* in cattle from the two districts according to age categories based on PCR and Giemsa‐stained blood smears. Bars, standard error; *, statistically significant by PCR; n.s, non‐significance showed by Giemsa.

Cattle belonging to the age group between 2 and 4 years were more infected by *T. annulata* than any other age group in both districts (40.47 ± 0.07 and 42.4 ± 0.08%, in Sidi Bouzid and Kasserine districts, respectively), with no statistically significant difference (*p* = 0.4) (Figure 2).

There was no statistically significant difference between the infection prevalence according to gender by both Giemsa‐stained blood smears and PCR (*p* = 0.2) (Table 1).

### 3.2 Phylogenetic relationship

**FIGURE 3:**
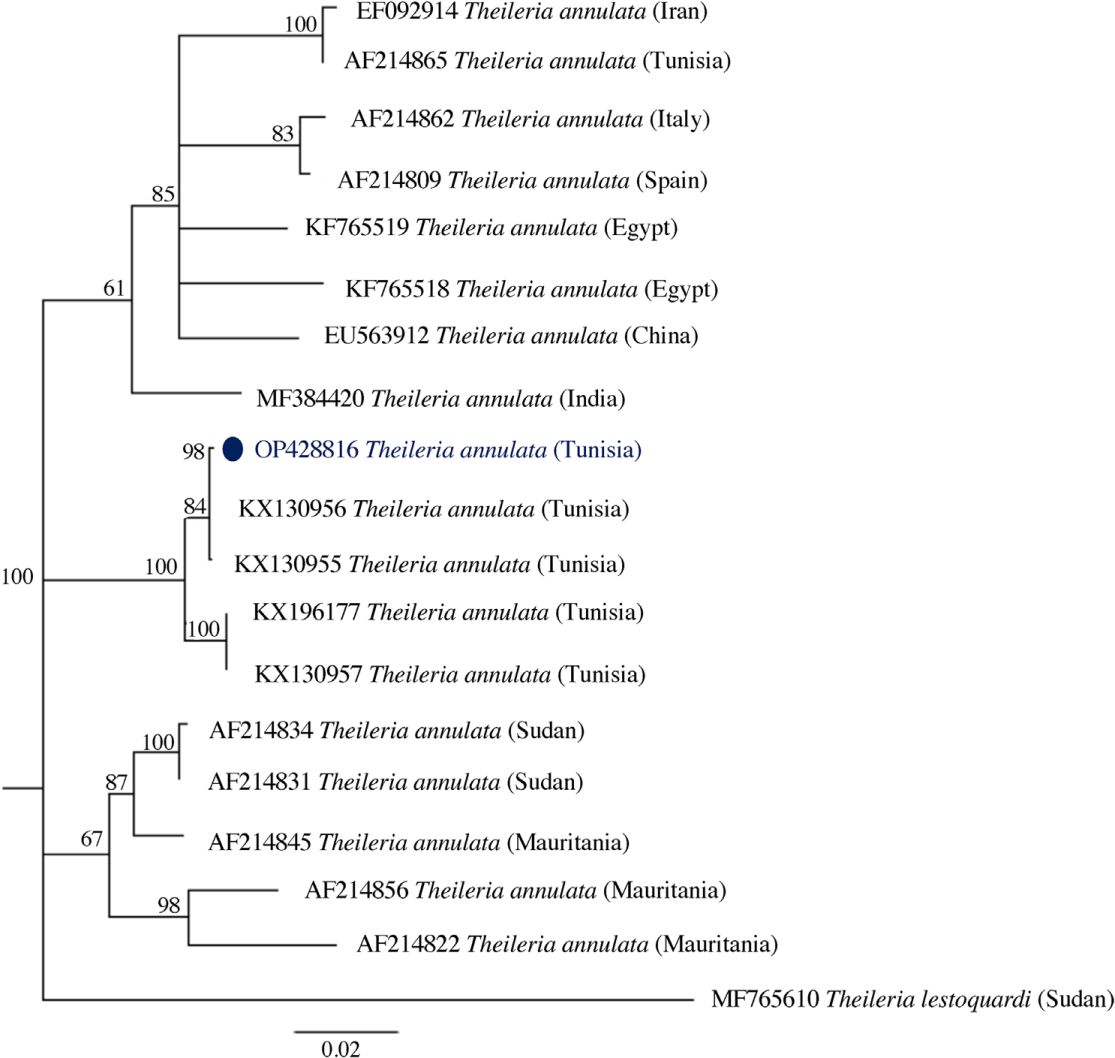
Phylogenetic tree using neighbour joining method showing the relationship between the Tunisian *Theileria annulata* Tams1 isolate (indicated with a blue dot) with available sequences in GenBank, *Theileria lestoquardi* was used as an outgroup.

Genetic variation among the *T. annulata* isolates in the two districts of central Tunisia was assessed using the Tams1 sequence. Phylogenetic analyses resolved the Tams1 sequences into three lineages with no relation to the geographic origin, except for one sequence (AF214865), which shares a 95% similarity with our sequence. All other Tunisian sequences belonged to the second lineage, with a similarity ranging from 97 to 100%. The Tunisian sequence isolated in this study shared 100% identity with a previously published Tunisian sequence isolated from North Tunisia (KX130956) and a 95% similarity with sequences from Mauritania (AF214856) and Sudan (AF214822) (Figure 3).

## 4 DISCUSSION

The present work aimed to estimate the infection prevalence of cattle by *T. annulata* in the Sidi Bouzid and Kasserine districts (central Tunisia) using Giemsa‐stained blood smears and PCR, their associated risk factors and to study the phylogenetic diversity of *T. annulata* strains in these two regions.

Out of 270 examined animals, 88 (32.6%) and 17 (6.3%) were detected as *T. annulata* carriers by PCR and Giemsa‐stained techniques, respectively. The sensitivity of PCR was significantly higher than that of the Giemsa‐stained technique (*p* < 0.001). The same trends were reported by Khattak et al. (2012), they reported carrier prevalence of 33 and 5.2% by PCR and Giemsa, respectively.

In Tunisia, TT clinical cases are reported mainly in the north, where *H. scupense* is abundant (Bouattour et al., 1996; Darghouth, 2000; Gharbi et al., 2014). For example, the recorded *T. annulata* infection prevalence in cattle was higher in Bizerte district (15.5%) using Giemsa (Boussaadoun et al., 2015) and in Ariana district (Hessiene region) using PCR (61%) (Sallemi et al., 2017). TT was also found in Gabes Oasis in the south‐east of Tunisia, with an infection prevalence of 26.5 and 31.2% by Giemsa and PCR, respectively (Gosrani, 1999).

In Algeria (Sétif district, Northeast), which is characterized by a semi‐arid climate favourable for the abundance of *H. scupense* tick vectors, a longitudinal survey was carried out and the infection prevalence of cattle by *T. annulata* was estimated at 16.4 and 50% using Giemsa and PCR, respectively (Ayadi et al., 2017). The higher prevalence observed in the present work compared to our study was associated with the sampling period, as they were collected after the tick season, which is a point that could be considered for future studies. The same trend was observed for all these studies using both techniques (Giemsa and PCR), and the difference could be related to the higher sensitivity of the PCR. For this reason, the use of blood smear examination is limited to diagnosing clinical cases of TT but not detecting carrier animals since they have very low parasitaemia (Darghouth et al., 2004; Gharbi et al., 2012).

Taha et al. (2013) estimated the infection prevalence of cattle by *T. annulata* in Northern Sudan, which has an arid climate, at 73.1%, which is higher than our results. This could be due to the abundance of *Hyalomma anatolicum* ticks in the studied area, which is another vector of *T. annulata*. Moreover, this study showed that 7.8% of tested sheep were infected by *T. annulata*. While in Tunisia, no study investigated the infection of sheep by this protozoan.

The visited farms had no history of TT clinical cases. Still, the cattle barns presented crevasses and cracks suitable for the development of the endophilic vector tick *H. scupense*, the main vector of this protozoan in Tunisia, which was not found on the animals in this study (Darghouth, 2000; Gharbi & Darghouth, 2014; Gharbi et al., 2014).

*Theileria annulata* infection was observed in all age groups, with no statistically significant difference between these groups. This finding surprisingly contrast results of previous studies on TT, which find a positive association between age and *Theileria* infection, where infection is higher in old animals due to repetitive exposure (Salih et al., 2007; Sallemi et al., 2017). However, infection was absent in animals of less than 1 year old. Probably due to the lower attractiveness of young cattle to ticks compared to adults, as ticks are attracted by volatile molecules released by the rumen, an organ that is either not functional or of small volume in young calves (Donzé et al., 2004). Several studies have documented that calves are approximately 70 times less attractive to ticks than adults (Bouattour et al., 1996; Gharbi, Hayouni et al., 2013; Stachurski, 1993).

There was no statistical difference between infection prevalence in female and male cattle. This agrees with previous studies that showed that gender is not a risk factor for *T. annulata* infection in cattle maintained in endemic stability farms since the tick populations are sufficiently high to reach animals of both sexes (Flach et al., 1995; Gharbi et al., 2014; Selim et al., 2022). However, the difference between the sexes is observed for clinical cases, as females develop more frequently TT clinical cases than males. This is due to the physiological status of females during lactation and pregnancy, which induces immunodepression.

All the surveyed cattle belonged to the Holstein breed, which is known to be more susceptible to *T. annulata* infection than indigenous and cross‐bred animals (Gharbi et al., 2014; Hussein et al., 2012). This was confirmed by using RNA‐seq to analyse the gene expression profile of *T. annulata*‐infected cells obtained from the Holstein breed compared to the local breed (Sahiwal). The transcriptome analyses showed significant differences in the expression level of host genes involved in parasite infection and oncogenesis between the two breeds, making the Holstein cattle more susceptible to *Theileria* infection than indigenous cattle in India (Larcombe et al., 2022).

Additionally, the sampling period can influence the *T. annulata* infection prevalence. The samples were collected in October from farms where TT clinical cases were never reported, while this disease is more prevalent in the summer which corresponds to the *H. scupense* activity season in northern Tunisia (Bouattour et al., 1999).

Indeed, a study performed by Bouattour (2009) highlighted the impact of environmental and climatic change on livestock farming, the vector, the pathogen and the animal reservoir or host constituting the vectorial system. The increase in an irrigated area in the district of Sidi Bouzid (45.6000 ha, i.e., 10% of the Tunisian irrigated areas) or even in other districts (e.g., the Gabes oasis) favours the establishment of the tick vector and subsequently the transmission of the parasite.

Moreover, global warming and the cohabitation of cattle with dromedaries are risk factors for the emergence of the dromedary's ticks *Hyalomma dromedarii*, which is a vector of *T. annulata* in Mauritania (d'Oliveira et al., 1997; Jacquiet et al., 1994). This tick species infests various domestic animals, mainly dromedaries (Elati et al., 2018; Gharbi et al., 2013) but also sheep (Rjeibi et al., 2016) and cattle (Gosrani, 1999) in Tunisian arid to Saharan bioclimatic zones. Nevertheless, it needs to be confirmed whether *H. dromedarii* is also a vector of *T. annulata* in Tunisian cattle since the engorged nymphs of this species were found on cattle sympatric with dromedaries, which were found in the Oasis of Hazoua (Southern Tunisia) (Hniche, 2006). If that is the case, it could lead to the spread of TT in a larger area, threatening a higher number of cattle populations, especially the exotic breeds, which are used in milk production and are more susceptible to TT than the indigenous breed, as explained above (Larcombe et al., 2022).

A phylogenetic study of the Tams1 gene in this region showed a similarity of 95 to 100% with strains from Northern Tunisia that were clustered into two groups. This confirmed a previous study by Sallemi et al. (2017), who found Tams1 to be diverse. The clustering of the Tams1 sequences into three lineages suggests the presence of three genotypes with no correlation with the geographic origin. The newly isolated sequence in the present study shares a 100% identity with previously published Tunisian sequences from the north of the country (KX130956) and a 95% similarity with sequences from Mauritania (AF214856) and Sudan (AF214822). This diversity confirms a previous finding where the phylogenetic analyses of Tams1 sequences from three continents resolved the sequences into two major groups where some isolates revealed a geographic specificity (Wang et al., 2014).

## 5 CONCLUSIONS

The data presented herein confirm that TT is also present in central Tunisia despite the absence of the *H. scupense* vector tick. An assessment and monitoring of TT in this region and other regions from the central Tunisia should be carried out regarding the medical and economic importance of this disease in the livestock industry. An investigation of the role of *H. dromedarii* in transmitting *T. annulata* in dry areas is paramount to avoid the spread of the disease.

An improvement of cattle barns is recommended, mainly plastering and smoothing their external and internal surfaces, accompanied by cleaning the cattle barns and their surrounding areas. This control measure should be associated with acaricide application on animals during the summer, targeting the adult ticks, and in autumn, targeting the immature stages (larvae and nymphs). Further, on small farms, the manual removal of ticks should be performed by farmers during cow milking. Vaccinating animals against ticks by injecting the protein Hd86 is a sustainable control option that reduces the direct pathogenic effects of ticks and annihilates their vector role (Galaï et al., 2012).

## AUTHOR CONTRIBUTIONS

Conception and design of the study: MG and KE. Acquisition of data: IS and RK. Analysis and/or interpretation of data: KE. Drafting the manuscript: KE. Revising and editing of the manuscript: KE, IS, RK, MG. All authors contributed to the article and approved the submitted version.

## CONFLICT OF INTEREST STATEMENT

The authors declare no conflict of interest in relation to this work.

## ETHICAL STATEMENT

Ethical concerns were taken into account by adhering to Tunisian animal welfare regulations and practices and conformed to ethical guidelines for animal usage in research of the National School of Veterinary Medicine of Sidi Thabet (Tunisia) and the Association Tunisienne des Sciences des Animaux de Laboratoire (ATSAL, Tunisia). Animals were handled in the presence of their owners by licensed veterinarian and blood collection is a routine veterinary manipulation.

## Data Availability

The data presented in the study consisting of unique Tams1 sequence is deposited in GenBank under the accession number OP428816.
